# Supplementary figures and images for: Direct mosquito feedings on dengue-2 virus-infected people reveal dynamics of human infectiousness
Source: PLoS Negl Trop Dis. 2023 Sep 1;17(9):e0011593. doi: 10.1371/journal.pntd.0011593 (PMC10501553; doi:10.1371/journal.pntd.0011593)

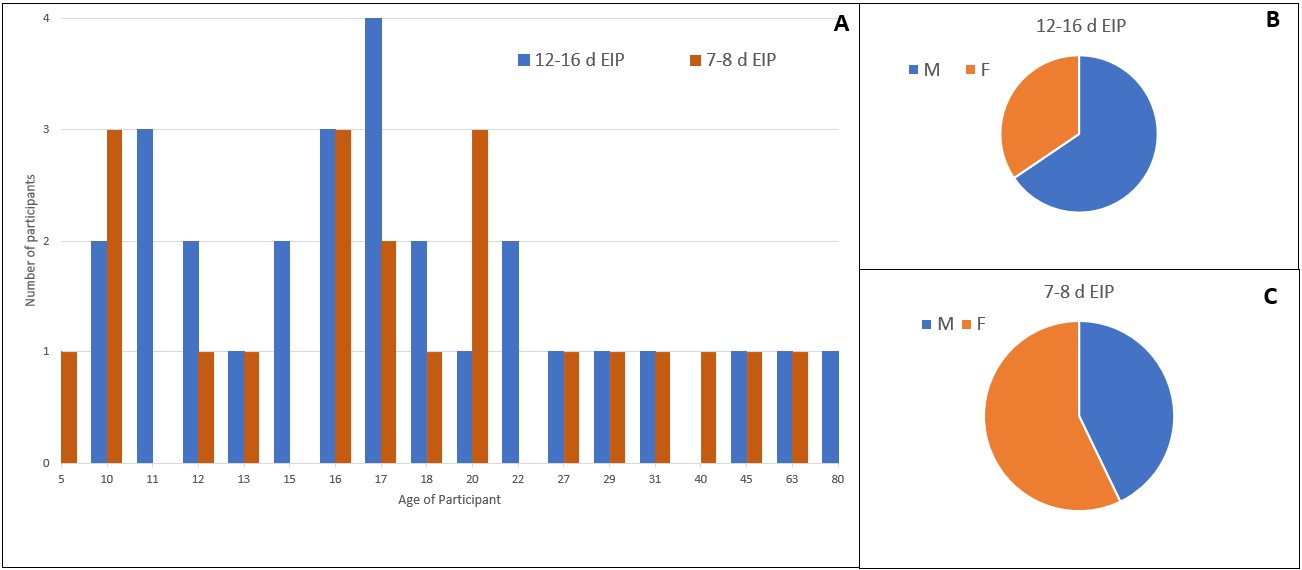

Supplement: S1 Fig — (TIF) [file pntd.0011593.s001.tif]
