# Supplementary material for: Direct mosquito feedings on dengue-2 virus-infected people reveal dynamics of human infectiousness
Source: PLoS Negl Trop Dis. 2023 Sep 1;17(9):e0011593. doi: 10.1371/journal.pntd.0011593 (PMC10501553; doi:10.1371/journal.pntd.0011593)
Supplement: S1 Text — Contains Figs A-B and Tables A-C. Fig A. Flow chart of model selection process. Fig B. Linear model fitting for day of illness. The plot shows the residuals of the linear fit against day of illness. Thin lines represent the 25th and 75th percentiles and solid lines represent the median. Table A. GLM with linear terms for each covariate (AIC = 163.2932). Table B. GAM with nonlinear smooth term for day of illness and linear terms otherwise (AIC = 146.0056). Eff. df: effective degrees of freedom; Ref. df: reference degrees of freedom. Table C. GLM with day of illness as a factor and linear terms otherwise (AIC = 149.6279). (DOCX) [file pntd.0011593.s003.docx]

**Supporting information (Model Selection)**

# ***Supplementary results***

**
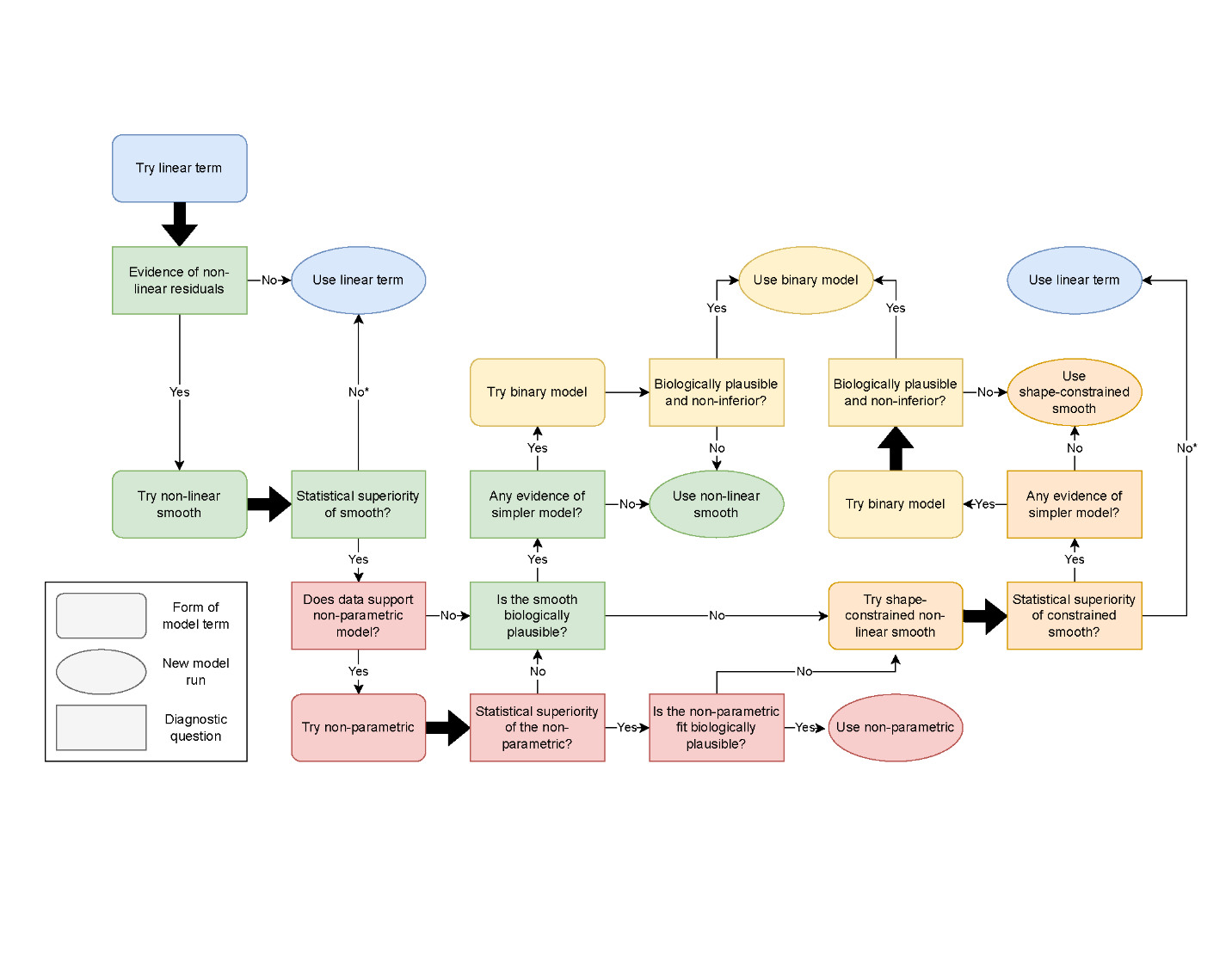
**In general, model selection regarding the functional form (or presence) of each covariate yielded identical results if done independently or simultaneously across covariates. Each permutation was investigated, but for brevity, we describe here the main model selection choices for each covariate. Below, when we refer to the ‘most complex smooth model’ considered, this is a model with shape constraints for viremia (non-decreasing) and extrinsic incubation time (non-decreasing), and a non-constrained smooth for day of illness. Fig S1 provides a flow diagram of the model selection process that was followed to fit each of the model terms.

**Fig A.** Flow chart of model selection process.

**Viremia**. Model selection on viremia was the simplest to conduct. Starting with the most complex functional form (a shape-constrained monotonic non-decreasing smooth), we found a statistically significant effect of viremia on the outcome (e.g., within the most complex smooth model: *p*-value = 0.000353), but the effective degrees of freedom were 1.006, suggesting that the relationship was very close to linear. The AIC of this model (421.13) was very close to both the AIC of the same model with an unconstrained smooth on viremia (421.08) as well as the same model with a linear term on viremia (421.13). It should be noted that the unconstrained smooth term happened to also be monotonically non-decreasing. Following our model selection protocol, we selected the ‘simplest’ functional form for viremia, which was the simplest we considered: linear.

**Day of illness**. Model selection for the functional form of day of illness was more complicated. The linear approach yielded a non-significant result (e.g., within the simplest linear term-only model: *p*-value = 0.741, Table S1). However, by investigating the residuals of the fit against day of illness (Figure S2), there is some suggestion that the model is underestimating the outcome on day 2 of illness. From here, there are two options: 1) a non-linear smooth term or 2) a non-parametric non-linear approach where each day of illness gets its own effect. Replacing the linear term for day of illness in the simplest linear term-only model with a non-linear smooth (and thus recasting the model as a generalized additive model [GAM]) yields a statistically significant non-linear effect of day of illness (*p*-value = 0.00394, effective degrees of freedom = 1.913, Table S2). Replacing the linear term for day of illness with day of illness as a factor and fitting a different effect for each day yields statistically significant effects for day 2 versus the reference of day 1 (*p*-value = 0.00369, Table S3). Days 3, 4, and 5 were not statistically significantly different from day 1 (*p*-values = 0.356, 0.994, and 0.996, respectively). In terms of model selection, the AIC of the GAM was slightly preferable to the non-parametric approach ($\Delta AIC=3.62$). As noted in the main text, this is a relatively small difference in AIC. Moreover, it could be argued that the non-parametric approach is simpler, even though it uses more degrees of freedom. Both models should be considered, and it is critical to note that both models have the same main feature: day of illness 2 results in higher predicted outcomes than day of illness 1 or 3. For the primary model discussed, we proceed with the non-linear smooth term on day of illness.

**Table A.** GLM with linear terms for each covariate (AIC = 163.2932).

| Model term | Estimate | Std. Error | z-value | *p*-value |
| --- | --- | --- | --- | --- |
| Intercept | -7.967 | 1.1246 | -7.085 | $1.4\times{10}^{-12}$ |
| Log viremia | 0.2758 | 0.0783 | 3.521 | 0.00043 |
| Day of illness | -0.0473 | 0.1428 | -0.331 | 0.7406 |
| Incubation time | 0.3429 | 0.0608 | 5.639 | $1.7\times{10}^{-8}$ |

**Fig B.** **Linear model fitting for day of illness.** The plot shows the residuals of the linear fit against day of illness. Thin lines represent the 25^th^ and 75^th^ percentiles and solid lines represent the median.

**Table B.** GAM with nonlinear smooth term for day of illness and linear terms otherwise (AIC = 146.0056). Eff. df: effective degrees of freedom; Ref. df: reference degrees of freedom.

| Model term | Estimate | Std. Error | z-value | *p*-value |
| --- | --- | --- | --- | --- |
| Intercept | -8.5857 | 0.9793 | -8.767 | $<2\times{10}^{-16}$ |
| Log viremia | 0.2604 | 0.0841 | 3.098 | 0.00195 |
| Incubation time | 0.3412 | 0.0614 | 5.558 | $2.7\times{10}^{-8}$ |
|  |  |  |  |  |
| Model term | **Eff. df** | **Ref. df** | **Chi^2^** | ***p*-value** |
| Day of illness | 1.913 | 1.992 | 10.5 | 0.00394 |

**Table C.** GLM with day of illness as a factor and linear terms otherwise (AIC = 149.6279).

| Model term | Estimate | Std. Error | z-value | *p*-value |
| --- | --- | --- | --- | --- |
| Intercept | -8.2714 | 1.0730 | -7.709 | $1.3\times{10}^{-14}$ |
| Log viremia | 0.25931 | 0.08475 | 3.060 | 0.00221 |
| Day of illness 2 | 0.85319 | 0.29386 | 2.903 | 0.00369 |
| Day of illness 3 | 0.37161 | 0.40228 | 0.924 | 0.35561 |
| Day of illness 4 | -17.3455 | 2502.20 | -0.007 | 0.99447 |
| Day of illness 5 | -17.9641 | 3505.86 | -0.005 | 0.99591 |
| Incubation time | 0.34032 | 0.06135 | 5.547 | $2.9\times{10}^{-8}$ |

**Extrinsic incubation**. As with viremia, starting with the most complex functional form (a shape-constrained monotonic non-decreasing smooth), we found a statistically significant effect of extrinsic incubation time on the outcome (e.g., within the most complex smooth model: *p*-value =$9.4\times{10}^{-7}$), but the effective degrees of were 1.000, suggesting that the relationship was very close to linear. The AIC of this model (421.13) was very close to both the AIC of the same model with an unconstrained smooth on incubation time (421.02), but substantially lower than the same model with a linear term on incubation time (431.97). Here, it should be noted that the unconstrained smooth term was not monotonically non-decreasing and predicted lower outcomes for 13 or more days of incubation than for 12 days of incubation. There is no obvious biological driver of this phenomenon, and this is most likely due to inherently smaller sample sizes for longer incubation times (as more mosquitoes will have died the longer the experiment lasts). Either way, it is clear from visual inspection of the data that the outcomes are very low for short incubation times and much higher for more than 11 days of incubation. As an intermediate analysis, we subsetted the data to only those experiments with more than 11 days of incubation. As both a linear term and a non-linear term, there was no significant effect of incubation time on the outcome (*p*-values of 0.447 and 0.344, respectively). Led by these analyses and the visual inspection indicating that there is a difference between incubation times less than 9 days or greater than 11 days, we assessed the effect of a stepped relationship of incubation time on the outcome (with a constant effect for incubation times less than 9 days and a separate constant effect for incubation times greater than 11 days). Again, comparing to the most complex functional form but substituting in a stepped effect of incubation time, we found not only a statistically significant effect (*p*-value = $4.7\times{10}^{-7}$), but a very comparable AIC to the more complicated functional forms (422.3). As this model is considerably simpler than the more complex functional forms, the stepped relationship was chosen for the final model.

## **Relationships between covariates**. While correlation between covariates can be captured within the various regressions in terms of the correlation between the coefficients of those covariates, it is useful to look more explicitly at these relationships when trying to predict the outcome. In particular, while our final model suggests that an individual may be more infectious on day 2 of their illness than day 1 for the same level of viremia, it is unclear if one would expect any individual to actually have similar levels of viremia on day 1 and day 2 of their illness.
