# Supplementary material for: Direct mosquito feedings on dengue-2 virus-infected people reveal dynamics of human infectiousness
Source: PLoS Negl Trop Dis. 2023 Sep 1;17(9):e0011593. doi: 10.1371/journal.pntd.0011593 (PMC10501553; doi:10.1371/journal.pntd.0011593)
Supplement: Attachment — Submitted filename: Editorial Office Responses.docx [file pntd.0011593.s004.docx]

Thank you for submitting your paper, "Direct mosquito feedings on dengue-2 virus-infected people reveal dynamics of human infectiousness", to PLOS Neglected Tropical Diseases. We have checked over your submission and, before we can assign your manuscript to the Editor, we ask that you first please make the following modifications:

1) Thank you for stating"Code and data are openly available on GitHub [link to be placed here upon acceptance] and Zenodo [link to be placed here upon acceptance], respectively".

We strongly recommend all authors deposit their data before acceptance, as the process can be lengthy and hold up publication timelines. Please note that, though access restrictions are acceptable now, your entire data will need to be made freely accessible if your manuscript is accepted for publication. This policy applies to all data except where public deposition would breach compliance with the protocol approved by your research ethics board. If you are unable to adhere to our open data policy, please kindly revise your statement to explain your reasoning and we will seek the editor's input on an exemption. Please be assured that, once you have provided your new statement, the assessment of your exemption will not hold up the peer review process.

Response: We have modified the above statement as follows:

“R code and data used for statistical analysis and to produce figures in the manuscript are openly available on GitHub [https://github.com/bcreiner/mosq_feeds_Iquitos] and provided as a supplementary file (S2_file) , respectively.”

Note the data set is now included as supplementary information at S2_file.

2) Please provide an Author Summary. This should appear in your manuscript between the Abstract (if applicable) and the Introduction, and should be 150–200 words long. The aim should be to make your findings accessible to a wide audience that includes both scientists and non-scientists. Sample summaries can be found on our website under Submission Guidelines:

<https://journals.plos.org/plosntds/s/submission-guidelines#loc-parts-of-a-submission>

Response: Sorry for that oversite, it is now included in manuscript file.

3) We notice that your Supplementary Figures (S1 Figure,S2 Figure) are duplicated on your submission as they are uploaded separately and are also part of your (S1_textfile_25July2023_submit.docx). Please remove them and upload them with the file type 'Supporting Information' leaving only the supplementary legends in the manuscript file and make sure that the current and most updated version of them are uploaded correctly and only once.

Response: We have now removed S1 Figure and S2 Figure as they are part of the S1 Text file, and have modified referrals in the text.

4) Thank you for stating"Written informed consent was obtained from all adult participants and the parents or guardians of all participants between 5-17 years".

Please be so kind and clarify the age limit of child participants.

NOTE: If child participants ["Below 18 years"] , the statement must declare that formal consent (must state whether written/verbal) was obtained from the parent/guardian.

Response: Our current ethics statement clearly does this:

“Written informed consent was obtained from all adult participants and the parents or guardians of all participants between 5-17 years. Participants that were minors provided assent (written for 8- to 17-years old, verbal for <8 years old).”

This clearly states that both adult participants as well as parents/guardians provide written consent, it is just in the sentence prior to describing the assent process.

Once you have made the necessary changes in Editorial Manager, please re-approve the submission by following the same steps as before.

Please resolve these issues and resubmit your manuscript within 30 days. If you need more time, please email the journal office at [plosntds@plos.org](mailto:plosntds@plos.org). We are happy to grant extensions of up to one month past this due date. If we do not hear from you within 30 days, we will withdraw your manuscript on your behalf.
